# Extraction and Identification of a Wide Range of Microplastic Polymers in Soil and Compost

**DOI:** 10.3390/polym13234069

**Published:** 2021-11-23

**Authors:** Franja Prosenc, Pia Leban, Urška Šunta, Mojca Bavcon Kralj

**Affiliations:** 1 Research Institute, Faculty of Health Sciences, University of Ljubljana, 1000 Ljubljana, Slovenia; urska.sunta@zf.uni-lj.si; 2 Department for Sanitary Engineering, Faculty of Health Sciences, University of Ljubljana, 1000 Ljubljana, Slovenia; pia.leban@ijs.si (P.L.); mojca.kralj@zf.uni-lj.si (M.B.K.)

**Keywords:** microplastic extraction, oil extraction, density separation, GC–MS, mass spectrometry identification, plastic polymers, polyethylene terephthalate, polyethylene, terrestrial

## Abstract

Microplastic pollution is globally widespread; however, the presence of microplastics in soil systems is poorly understood, due to the complexity of soils and a lack of standardised extraction methods. Two commonly used extraction methods were optimised and compared for the extraction of low-density (polyethylene (PE)) and high-density microplastics (polyethylene (PET)), olive-oil-based extraction, and density separation with zinc chloride (ZnCl2). Comparable recoveries in a low-organic-matter matrix (soil; most >98%) were observed, but in a high-organic-matter matrix (compost), density separation yielded higher recoveries (98 ± 4% vs. 80 ± 11%). Density separation was further tested for the extraction of five microplastic polymers spiked at different concentrations. Recoveries were >93% for both soil and compost, with no differences between matrices and individual polymers. Reduction in levels of organic matter in compost was tested before and after extraction, as well as combined. Double oxidation (Fenton’s reagent and 1 M NaOH) exhibited the highest reduction in organic matter. Extracted microplastic polymers were further identified via headspace solid-phase microextraction–gas chromatography–mass spectrometry (HS-SPME–GC–MS). This method has shown the potential for descriptive quantification of microplastic polymers. A linear relationship between the number of particles and the signal response was demonstrated for PET, polystyrene (PS), polyvinyl chloride (PVC), and PE (R^2^ > 0.98 in alluvial soil, and R^2^ > 0.80 in compost). The extraction and identification methods were demonstrated on an environmental sample of municipal biowaste compost, with the recovery of 36 ± 9 microplastic particles per 10 g of compost, and the detection of PS and PP.

## 1. Introduction

Microplastic (MP) pollution is widespread across all ecosystems, and has been widely studied in marine systems, whereas other environmental compartments—such as soil—have only started to emerge as a field of research in the past few years [1,2]. Soil is a very versatile and complex matrix with a broad range of organic matter content (from 0.02% in desert soils to up to 100% in bog soils) [2]. Specifically, the high organic matter content of soils hampers the extraction of MPs due their having similar densities to most common plastic polymers and, as such, poses a challenge in the determination of MPs in soils. Recently, standards for the collection and preparation of water samples for the identification and quantification of MP particles and fibres have been developed (ASTM D8332-20 and D8333-20) [3,4]. However, to date, there are no such standards for soil and soil-like samples, even though various methods for the detection of MPs in soil have been proposed.

MPs enter soil via the addition of soil amendments—such as biowaste compost and waste sewage sludge/biosolids—the use of agricultural plastics, irrigation with (reclaimed) wastewater, flooding, and atmospheric deposition, as well as littering and street runoff [2,5,6,7]. Studies reporting on primary sources of MPs in soil consider tire wear, fibres from synthetic clothing, artificial turf, and agricultural plastics (plastic mulch films, greenhouses, fruit protection foams, etc.) as notable sources [8,9,10]. Agricultural soils are particularly prone to accumulating MPs [11]. A study quantifying MPs in soil reported between 7100 and 42,960 plastic particles per kilogram of soil in southwestern China, 95% of which were <1 mm [12]. A study in northwestern China found 40 ± 126 light-density polyethylene (LDPE) MPs per kilogram of soil in the top 10 cm of agricultural soil, and 100 ± 141 LDPE MPs per kilogram of soil in the 10–30 cm layer [13]. In east China, a study found 40.2 ± 15.6 MPs per kilogram of unamended agricultural soil, while in soil amended with sludge, between 68.6 ± 21.5 and 149.2 ± 52.5 MPs per kilogram of soil were found [14]. In Germany, 0.34 ± 0.36 MPs were found per kilogram of dry weight of soil that had never been fertilised with MP-containing fertilisers [15]. Organic waste from households that is composted, or anaerobically digested with further composting of digestate, is usually applied to soil as a fertiliser. This is a common practice to return nutrients, trace elements, and humus to the soil; however, most municipal organic waste is contaminated with plastics—either with non-biodegradable plastic bags and/or other plastic items [16]. A study by Weithmann et al. reported between 20 and 24 MP particles per kilogram of dry weight biowaste compost, and between 14 and 146 MPs per kilogram of dry weight biowaste digestate [17].

MPs in soil have been shown to have deleterious effects on soil organisms; however, most studies were carried out at (currently) environmentally irrelevant concentrations. These high concentrations, however, represent future, presumably higher levels, as plastic production, use, and consequent pollution increase [18]. MPs have been shown to alter the microbial activity in soil and sediments, and to increase root and shoot mass [19,20], affect the mortality and growth rate of earthworms (*Lumbricus terrestris*) [21], affect the reproduction of nematodes (*Caenorhabditis elegans*) [22], and alter the immune response in crustaceans (*Porcellio scaber*) [23]. In addition to the biotic effects of MPs, they have also been shown to influence soils’ biophysical properties, such as pH, content and size distribution of water-stable aggregates, water-holding capacity, soil bulk density, and saturated hydraulic conductivity [19,24,25,26].

To precisely evaluate the risks of MP pollution in agroecosystems and terrestrial environments, reliable methods for the identification and quantification of MPs are needed. Extraction of MP particles from these complex matrices is a paramount step in their analysis. To date, various methods for the extraction of MPs have been developed, including density separation, oil extraction, electrostatic separation, magnetic-field separation, solvent extraction, and circular separation [27,28,29,30,31]. Density separation using various saturated saline solutions—such as sodium iodide (NaI), sodium chloride (NaCl), sodium bromide (NaBr_2_), calcium chloride (CaCl_2_), zinc bromide (ZnBr_2_), and zinc chloride (ZnCl_2_)—is the most frequently applied extraction method for solid samples. In this method, MP particles are separated from the solid matrix based on the difference between the density of MPs and the density of the saturated saline solution. Various technical solutions have been used to separate MP-containing supernatants without disturbing the settled sediment. Most commonly, the supernatant is decanted onto a filter after a settling period, then withdrawn with a pipette or sucked with the aid of a pump [13,32]. Imhof et al. [33] devised the “Munich Plastic Sediment Separator”—a custom-made stainless steel apparatus that separates the settled sediment from the MP-containing supernatant with a valve. Konechnaya et al. [34] separated MP-containing supernatant from various sediments by slowly overflowing the samples with saline solution and collecting the MP-containing overflow. Recently, Grause et al. [35] proposed using a centrifuge to speed up the separation step and decanting the supernatant with MPs onto a filter. Many of these methods, however, were not tested on soils or organic-rich matrices, such as compost.

Oil-based separation has recently been developed, with promising applications. In this method, MPs are extracted based on the oleophilic properties of most plastic polymers, so that the MPs, when in contact with oil, move to the oil layer independent of their density. Water and a small volume of oil are added to the solid sample, stirred, and left to settle, and then the top oil layer, containing MPs, is separated from the rest of the sample. In several studies, a separation funnel was used to drain the sediment and aqueous fraction from the sample and, in this way, retain the MP-containing oil layer [27,36,37]. Several types of oil—such as canola, castor, and olive oils—were used for the separation of MPs from sediments, soil, and sludge. It has been reported, however, that separation funnels could easily become obstructed when extracting solid samples [36]. The same issue was encountered in our preliminary experiments. Scopetani et al. [38], therefore, designed a system to freeze the samples after the settling period and cut off the frozen oil layer.

After extraction, MPs can be identified by employing advanced instrumentation, using non-destructive (A) and destructive methods (B): (A) scanning electron microscopy (SEM) combined with spectroscopic techniques, such as micro-Fourier-transform infrared (µFTIR) and Raman spectroscopies; (B) thermogravimetric analysis (TGA), thermal desorption gas chromatography coupled with mass spectrometry (TED-GC–MS), and pyrolysis employing gas chromatography coupled with mass spectrometry (Py-GC–MS) [39,40,41,42,43]. Even though the well-established methods can offer enhanced identification of polymers, the field strives towards developing low-cost and simple techniques to facilitate analysis in most laboratories with basic analytical equipment. For this reason, solid-phase microextraction (SPME)—as a simple, inexpensive, and easy-to-handle technique—combined with gas chromatography coupled with mass spectrometry (GC–MS) analysis was used to develop an efficient method for the identification of MPs in environmental matrices, termed headspace solid-phase microextraction–gas chromatography–mass spectrometry (HS-SPME–GC–MS) [44,45].

This study aimed to determine the most suitable method for the extraction of various MP polymers from soil and compost, based on recovery, ease of handling, the potential for operator error, and time efficiency. Two methods—an olive-oil-based method, and density separation with saturated ZnCl_2_ solution, never compared before—were evaluated for the extraction of MPs. Moreover, the effects of different vessels on MP recovery with an oil-based method were evaluated for the first time. Additionally, a simpler identification method based on HS-SPME–GC–MS is presented in this study as a viable alternative to traditionally used identification methods, and shows the potential for descriptive quantification of MPs. This method allowed for the simultaneous identification of various polymers in a mixture, and showed good linearity by increasing the number of spiked MPs in real matrices (both alluvial soil and compost), which is a significant advantage over the traditionally used methods.

## 2. Materials and Methods

### 2.1. Preparation of Microplastics

**Figure 1:**
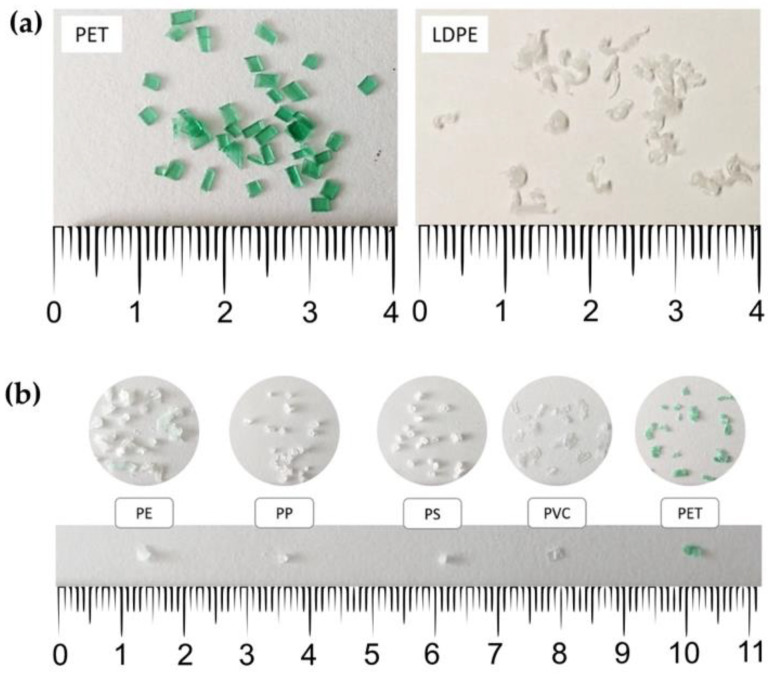
Microplastic particles prepared for (**a**) extraction method optimisation and (**b**) extraction of a wide range of microplastic polymers (PP: polypropylene; PE: polyethylene; PS: polystyrene; PVC: polyvinyl chloride; PET: polyethylene terephthalate).

Two types of MP polymers were used in the development and optimisation of the extraction methods—polyethylene terephthalate (PET), as a polymer with high density (ρ = 1.33–1.48 g cm^−3^), and low-density polyethylene (LDPE), as a polymer with low density (ρ = 0.91–0.94 g cm^−3^) (Figure 1a). PET particles were prepared by cutting a plastic bottle (Radenska, Radenci, Slovenia) into <5 mm pieces. LDPE particles were prepared by melting plastic pellets (Tera Tolmin, Ltd., Tolmin, Slovenia), and then grating the melted plastic mass using a metal cheese grater and sieving the particles with a 2 mm sieve (Retsch, Dusseldorf, Germany).

After the method optimisation, the method was tested on five MP polymers—namely, polypropylene (PP), polyethylene (PE), polystyrene (PS), polyvinyl chloride (PVC), and PET (Table 1, Figure 1b). The same particles were then identified via HS-SPME–GC–MS. The MP particles were prepared with cryo-milling according to an adapted protocol [44]. In short, 5–10 g of plastic material (pellets or small pieces) was added to a stainless-steel grinding jar, together with a stainless steel 25 mm grinding ball. The jar was submerged in liquid nitrogen for 6 min, after which the grinding was carried out in a MillMix 20 ball mill (Tehtnica, Železniki, Slovenia) at between 25 and 30 Hz for 1.5 min, depending on the polymer. For PET and PE, the procedure of cooling and grinding was repeated more than once; the fine fraction was then sieved through 2, 1, and 0.5 mm sieves (Retsch, Germany) to obtain a fine fraction of 1–2 mm.

### 2.2. Optimisation of Microplastic Extraction

Two methods for the extraction of MPs from soil were adapted from the literature and optimised: olive-oil-based extraction, and density separation with saturated ZnCl_2_ solution [34,38]. Both methods were tested on two types of MP polymers: LDPE, representing MPs with low density; and PET, representing MPs with high density. In both methods, 10 and 20 MP particles of each composition were spiked into 10 g of alluvial soil or biowaste compost. Alluvial soil used in this experiment was collected on the banks of the Sava River basin (15°31′47.65″ E, 45°55′27.21″ N), in the immediate vicinity of agricultural fields, at a depth of 0–10 cm. The compost was from a biowaste composting plant that collects and processes separately collected organic waste (JP VOKA Snaga Ltd., Ljubljana, Slovenia), with a reported organic matter content of 24–44%.

#### 2.2.1. Oil-Based Method

**Figure 2:**
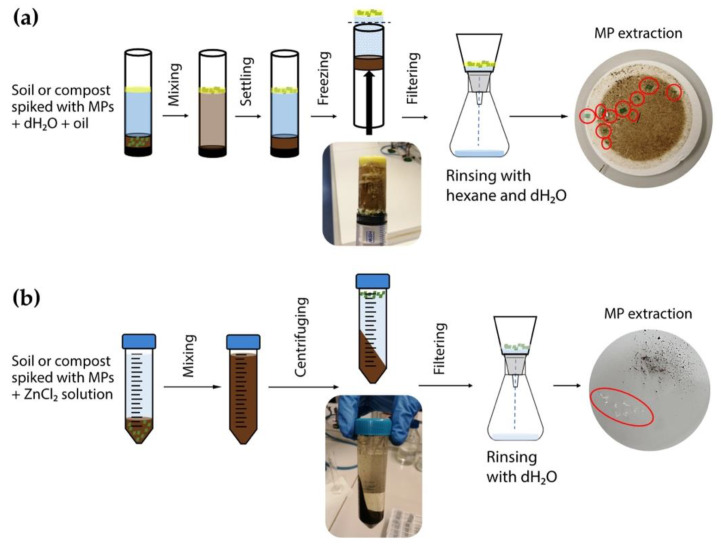
Schematic representation of microplastic extraction methods: (**a**) oil-based method, and (**b**) density separation.

This method was carried out as described by Scopetani et al. [38], with some modifications, as shown in Figure 2a. As a vessel for the sample, three technical configurations were used: a polytetrafluoroethylene (PTFE or Teflon) cylinder (Dastaflon Ltd., Medvode, Slovenia), a glass cylinder (Promal, Logatec, Slovenia), and a modified plastic syringe with the hub removed to create an open, flat aperture (Soft-Ject^®^, Henke-Sass, Wolf GmbH, Tuttlingen, Germany). Ten grams of alluvial soil was spiked with either 10 or 20 LDPE or PET MPs added to each sample vessel and closed on one end with a cap. Then, 30 mL of dH_2_O was mixed with 3 mL of olive oil. Afterwards, the vessels were capped on the other end and shaken to homogenise the sample. Samples were left to stand for 2 h to sediment, and then frozen overnight at −18 °C. After freezing, the frozen samples were pushed out of the vessels with a piston. The frozen oil layer containing MPs was removed, melted at room temperature, and filtered through a 47 mm GF/C filter (Whatman, Maidstone, UK). The MPs and remaining debris were rinsed with hexane (CARLO ERBA Reagents S.A.S., Val-de-Reuil, France) and dH_2_O to remove any remaining oil. In the case of compost, the procedure was similar to that for alluvial soil, with slight modifications; the method was optimised using 10 PET MPs in a plastic syringe, and the oxidation step was added after extraction to reduce organic matter content. Oxidation was achieved by submerging the GF/C filter with the sample remaining on the filter in 60 mL of Fenton’s reagent for 2 h under constant stirring [46]. The oxidised sample was again filtered through the GF/C filter. Extraction of MPs in each treatment (vessel, MP type, and concentration) was repeated six times. Recovery of MPs was calculated using the following equation:

(1) $$R\;\left(\%\right)=\frac{N_{extracted}}{N_{spiked}}\times 100$$

#### 2.2.2. Density Separation

This method was carried out as described by Konechnaya et al. [34], with some modifications (Figure 2b). Saturated ZnCl_2_ solution with a density of 1.6 g cm^−3^ was prepared by dissolving 1 kg of ZnCl_2_ (anhydrous RE, CARLO ERBA Reagents S.A.S., Val-de-Reuil, France) in 751.4 mL of dH_2_O. The solution was adjusted to a pH of 3 with 5 M KOH, and then filtered through a 47 mm GF/C filter (Whatman, Maidstone, UK). Then, 10 g of alluvial soil was spiked with either 10 or 20 LDPE or PET MPs, and added to a 50 mL centrifuge tube. ZnCl_2_ solution was added to the 50 mL mark in the tube, and the sample was shaken for 30 s. The separation was carried out by centrifuging the sample at 9000 rpm for 15 min (Hettich Universal 320 centrifuge, Andreas Hettich GmbH & Co. KG, Tuttlingen, Germany). The supernatant, together with floated MP particles, was filtered through a GF/C filter. The filtrate—used ZnCl_2_ solution—was reused up to 20 times, since its density did not change. In the case of compost, the procedure was similar to that for alluvial soil, with slight modifications; the method was optimised using 10 PET MPs, and an oxidation step was added after extraction to reduce organic matter content. Oxidation was achieved by submerging the GF/C filter with the sample remaining on the filter in 60 mL of Fenton’s reagent for 2 h under constant stirring [46]. The oxidised sample was again filtered through the GF/C filter. Extraction of MPs in each treatment (MP type and concentration; matrix) was repeated six times. Recovery of MPs was calculated using Equation (1).

### 2.3. Extraction of a Wide Range of Microplastic Polymers

The more optimal of the two extraction methods (i.e., better recovery, easier to handle, less time consuming) was tested on MPs of five different polymer compositions—namely, PE, PP, PS, PVC, and PET (Table 1)—spiked into alluvial soil and compost. MPs were spiked into 10 g of matrix in different concentrations—namely, 10 MPs (2 particles of each polymer composition), 25 MPs (5 particles of each composition), and 50 MPs (10 particles of each composition). The extraction procedure was carried out as described in Section 2.2.2. Treatment of compost samples included double oxidation—one before extraction, and another after extraction. Before extraction, the whole sample (10 g of compost with MPs) was added to 60 mL of Fenton’s reagent and left for 2 h under constant stirring. Afterwards, the sample was filtered through the GF/C filter, and the sample remaining on the filter was oxidised again, this time using 50 mL of 1 M NaOH under constant stirring overnight at 50 °C. After the oxidation, the sample was filtered through a 100 µm stainless steel mesh (Fipis, Ribnica, Slovenia). The experiment was conducted in four replicates. Recoveries were calculated using Equation (1), and identification of cumulative extracted MPs was done using the HS-SPME–GC–MS method, as described in Section 2.4.

### 2.4. Identification of Microplastic Polymers Using Headspace Solid-Phase Microextraction with GC–MS

MP polymers from alluvial soil and compost samples were identified via the HS-SPME–GC–MS method described by Šunta et al. [44], with some modifications. The HS-SPME–GC–MS method is based on the adsorption of volatile compounds emitted during melting of plastic particles onto SPME fibre, followed by GC–MS analysis and detection of characteristic fragment ions for individual polymers (PET—*m*/*z* 163; PS—*m*/*z* 104; PVC—*m*/*z* 91; PP—*m*/*z* 142; and PE—*m*/*z* 85). Due to observed difficulties in the melting of PET in polymer mixtures, the method was optimised in the steps of sample preparation and thermal decomposition. In the sample preparation step, PET MPs were separated from other MPs and analysed separately. In the thermal decomposition step, headspace vials were placed in a sand bath with a temperature of 260 °C (probe thermometer, Amarell GmbH & Co. KG, Kreuzwertheim, Germany) for 15 min and 3 min for melting of PET MPs and other MP polymers, respectively. With this improved protocol, identification of MP polymers extracted from spiked samples of alluvial soil and compost was carried out in cumulative samples composed of three replicates. A linear relationship between the number of MP particles and the signal response, with the area under the chromatographic peak being the measure, was determined using the coefficient of determination (R^2^).

## 3. Results and Discussion

### 3.1. Comparison of Microplastic Extraction Methods

The two most widely reported methods for the extraction of MPs from soil and soil-like matrices—oil-based extraction, and density separation—were optimised for MP polymers of high (PET) and low density (LDPE) and compared. As per the recommendations of Scopetani et al. [38], olive oil was used for the separation of oleophilic MPs from the matrix in the oil-based method. Olive oil supposedly has the strongest affinity for a wide range of plastic polymers [38]. Different solutions to separate MPs from the matrix with the oil-based method are reported in the literature—from a custom-made PTFE cylinder with a piston, to separation funnels [27,37,38]. Separation funnels were tested, and were found to be impractical for the extraction of MPs from soil and compost, due to frequent clogging. This problem was also encountered by other researchers [36]. Three vessels were tested for the oil-based method: a PTFE cylinder, similar to the one used by Scopetani et al. [38]; a glass cylinder; and a modified plastic syringe. The glass cylinder was used as a cheaper alternative to the PTFE cylinder (i.e., EUR 15 vs. EUR 48 per piece), while the plastic syringe is a cheap and widely available laboratory consumable. Recoveries obtained in alluvial soil for all three vessels were high (>97%), and comparable for all spiked MPs (10 and 20 PET MPs, as well as 10 and 20 LDPE MPs), Table 2. The glass cylinder, however, was found to break frequently—not in the stages of freezing the sample, but rather when pushing the sample out after the freezing process. In compost, the recovery obtained for 10 PET MPs in a modified syringe was lower, at only 80%. It should be noted that in compost, only marginal conditions were tested—that is, using the lowest quantity of spiked MPs (10 MPs per 10 g of matrix) as well as the MPs with the highest density (PET), which are usually more challenging to separate. The recoveries achieved in this study for high-density MPs in soil were comparable to those obtained by Scopetani et al. [38], who used the extraction system most similar to ours (98% vs. 95%, respectively); for low-density MPs, however, the recoveries achieved were higher in this study (98% vs. 90%). In compost, Scopetani et al. [38] observed lower recovery of low-density MPs (PE and polyurethane (PU)) (80%), although the recovery of medium- and high-density MPs did not seem to be affected by the matrix. In this study, only high-density MPs (PET) were tested in the compost matrix, and their recovery was lower as compared to recovery in soil (80% vs. 98%, respectively). Other studies reporting on the use of the oil-based method used different solutions for extraction systems, e.g., separation funnels. Mani et al. [27] used separation funnels and castor oil, and achieved an average recovery of 99% of four polymers spiked into fluvial suspended surface solids, marine suspended surface solids, marine beach sediments, and agricultural soil; the matrix did not significantly influence MP recovery; however, the tested matrices were low in organic matter content and, therefore, less challenging. Crichton et al. [37] mixed samples with water and oil in an Erlenmeyer flask, and transferred the liquid fraction into a separation funnel during the sedimentation period. They achieved an average recovery of 96% of five polymers spiked into sediment beach samples.

The oil-based method did not prove to be straightforward in this experiment. MPs were often found to be stuck to the inner walls of vessels, as well as to the surface of the frozen samples. This was due to the formation of a thin layer of oil on the walls of the vessels, which the MPs were attracted to. To combat this, the inner walls were rinsed with hexane; after the sample was removed, the surface of the frozen sample was scraped, and these fractions were further processed to isolate MPs. This method was also time-consuming (2 h for settling, overnight for freezing, and up to 45 min for melting the cut-off sample containing MPs for further processing, e.g., filtration).

The second method tested was density separation with saturated ZnCl_2_ solution. This method followed previously published protocols, with further optimisation [32,33,34]. Sedimentation (usually overnight) was sped up with centrifugation (15 min). This significantly improved time efficiency, and improved decanting of the supernatant due to more compact sediment. Recoveries obtained in alluvial soil and compost were high (>93%, most >98%, respectively), and were comparable for all spiked MPs (different compositions and quantities) (Table 2). Additionally, the ZnCl_2_ solution was reused up to 20 times without losing the desired density, which significantly reduced the cost and the environmental footprint of the method. Density separation achieved better recoveries in compost as compared to the oil-based method (98% vs. 80%), with an oxidation step after filtration in both methods (see Section 2.2.1 and Section 2.2.2). For this method, oxidation with Fenton’s reagent before extraction was also tested, and the recovery obtained was 100% (*n* = 6). In the study by Konechnaya et al. [34], the authors used ZnCl_2_ solution and separated MPs from sandy matrices by overflow, and the obtained recoveries were comparable to those obtained in this study in alluvial soil (between 94 and 104% vs. 98%, respectively). Very recently, Grause et al. [35] used a similar approach to the one used in this study—density separation sped up via centrifugation using CaCl_2_ solution to extract MPs from agricultural soil. They optimised the centrifugation protocol on PET MPs spiked into soil, and obtained 95% recoveries, which is consistent with the results of our study; their method, however, was not tested on samples rich in organic matter; likewise, CaCl_2_ solution with a density of 1.4 g cm^−3^ could be unsuitable for the extraction of some MP polymers, e.g., some PET, chlorinated polyvinyl chloride (CPVC), polyvinylidene chloride (PVDC), etc. [35].

In addition to better recoveries in compost, the significant advantage of the density separation method over the oil-based method was in better time efficiency (approximately 30 min to obtain MPs on the filter vs. approximately 1 day, respectively), as well as in the ease of handling. The potential for scaling up—e.g., analysing multiple-kilogram samples—could be feasible with the use of a large-volume centrifuge. For this reason, density separation was further used in testing the method on a wide range of plastic polymers at various concentrations, as described in Section 3.2.

### 3.2. Extraction of a Wide Range of Microplastic Polymers

**Figure 3:**
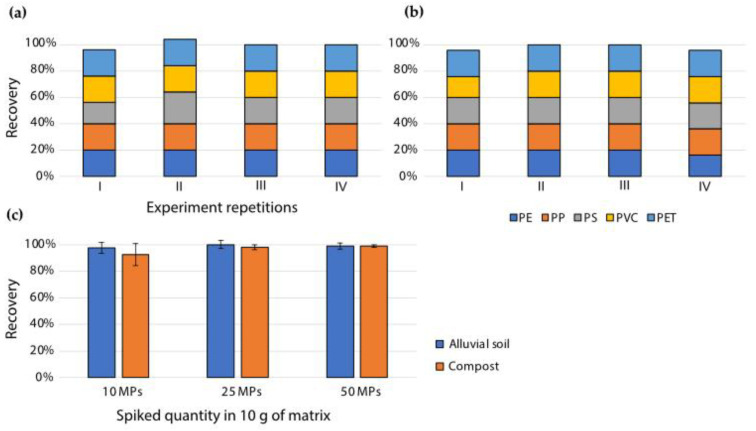
Recovery of MPs (polymer types: PE, PP, PET, PVC, and PS) spiked into alluvial soil and compost, extracted via the density separation method. Recovery of individual polymer types from (**a**) alluvial soil and (**b**) compost, spiked at a concentration of 5 MPs of each polymer type. (**c**) Cumulative recovery of all MPs, spiked at different concentrations. *n* = 4; results are presented as mean ± SD.

The extraction method that proved to be more optimal—density separation—was used for the extraction of MP particles of different polymer compositions (Table 1) spiked into alluvial soil and compost, at various concentrations. The five chosen MP polymers (PE, PP, PET, PVC, and PS) were chosen based on their frequent occurrence in the environment [15,17,47]. Cumulative recoveries were high (>93%) for all spiked MP polymers, especially at spiking quantities of 25 and 50 MPs (>98%) per 10 g of matrix (Figure 3c). Moreover, there were no significant differences in the recoveries of individual MP polymers from soil (Figure 3a) and compost (Figure 3b), nor were there any significant differences in recoveries from the two matrices. Blank samples of non-spiked alluvial soil were treated in the same way as the spiked samples to check for potential cross-contamination from aerial deposition, clothing, and/or lab equipment. Blank alluvial soil resulted in zero recovered MPs (*n* = 3). Blank compost samples were also treated in the same way; however, compost originating from the industrially composted organic fraction of household waste inherently contains plastic contamination from plastic bags and other sources [17]. A total of 32 ± 2 native MPs (*n* = 3) were recovered from the compost blank sample; however, their authenticity was not additionally checked with identification methods or the hot needle test. Native MPs did not hinder the determination of recovery of spiked MPs, since the spiked MPs were easily visually distinguishable. This method was additionally tested by inexperienced operators—primary school pupils, 14 years of age, with no prior laboratory experience; the recovery achieved ranged from 75% to 95%, which shows that the method is simple for handling.

**Figure 4:**
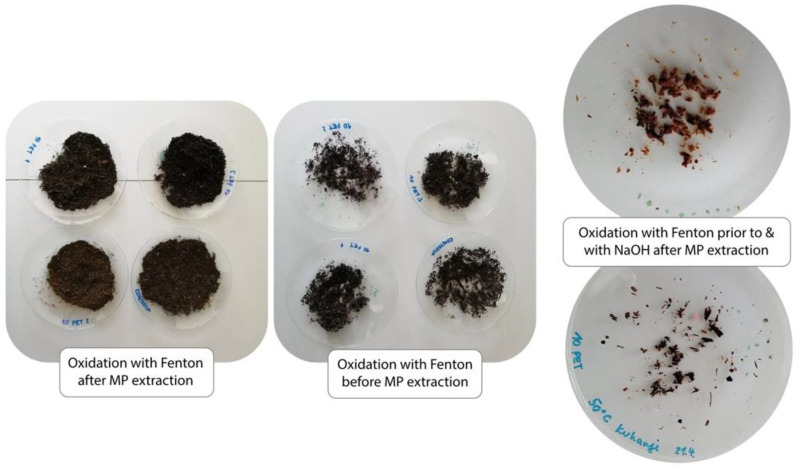
Reduction in organic content in compost samples with oxidation at various stages of the MP extraction procedure. The initial amount of all samples was 10 g of compost.

There are numerous reports on using density separation to extract various MPs from river and sea sediments; however, fewer studies have dealt with extraction from agricultural soil and organic-rich matrices, such as compost, which are considerably more difficult to process. Hurley et al. [46] tested a density separation protocol with NaI solution and an additional oxidation step on soil and sludge samples spiked with PE microbeads and PET fibres. Recoveries obtained in the reported study ranged between 92% and 100% for PE microbeads, and between 79% and 86% for PET fibres. The recoveries of PE microbeads resembled the recoveries obtained in this study (>92% vs. >93%, respectively); however, PET fibres were recovered to a lesser extent than the PET particles used in this study (>79% vs. 100%, respectively). Hurley et al. also tested the effect of an additional oxidation step on recoveries, and found that oxidation with Fenton’s reagent after MP extraction led to lower recoveries than oxidation before MP extraction, but this result was not statistically significant. No such effect was observed in this study; instead, we observed a difference in the quantity of organic matter removed (Figure 4). Liu et al. [31] devised a circulation separation device and tested various saline solutions to extract a wide range of MP polymers (polyamide (PA), polycarbonate (PC), PP, acrylonitrile butadiene styrene (ABS), PE, PS, poly(methyl methacrylate) (PMMA), polyoxymethylene (POM), PET, and PVC) from soil; with the use of NaBr and CaCl_2_, they achieved recoveries from 95% to 100%, similar to the recoveries reported herein. Similarly, Li et al. [48] constructed a circulation system for the extraction of MPs from soil via density separation using NaBr solution; the system was tested on common MP polymers (LDPE, PS, PP, and PVC) as well as biodegradable MPs (polybutylene succinate (PBS), poly(adipic acid), butylene terephthalate (PBAT), and polylactic acid (PLA)); recovery rates ranged from 92% to 99.6%, resembling the recoveries reported herein. Grause et al. [35], using density separation with centrifugation and CaCl_2_ solution, achieved recoveries between 97% and 98% for LDPE, PP, PS, and flexible PVC MPs, comparable to the recoveries obtained in this study; PET had lower recoveries as compared to the other tested MPs, at around 95%, whereas in this study no differences in recoveries of the five tested MP polymers were observed.

An oxidation step was employed for all compost samples in order to reduce the organic matter content and facilitate the identification of MP polymers. Oxidation was implemented at various stages of the MP extraction process to compare the efficiency of organic matter reduction. Oxidation with Fenton’s reagent after MP extraction reduced the organic matter content to a lesser extent compared to oxidation with Fenton’s reagent before MP extraction, as shown in Figure 4. Additionally, double oxidation was trialled—with Fenton’s reagent before extraction, and 1 M NaOH after extraction. Double oxidation reduced organic matter content to the largest extent (Figure 4), and was therefore used in processing all compost samples used in the recovery experiments. Hurley et al. [46] tested the impacts of different oxidation protocols on MP integrity; Fenton’s reagent proved to reduce organic matter to the highest degree (106%) without damaging the MPs; alkaline digestion with 1 M NaOH, on the other hand, provided a lesser reduction in organic matter (68%), but it also did not cause significant changes in the mass or size of MP particles [46].

### 3.3. Identification of Microplastic Polymers in Compost and Alluvial Soil

**Figure 5:**
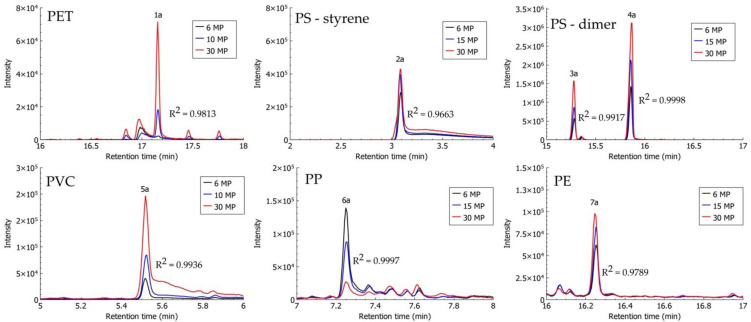
Headspace solid-phase microextraction–gas chromatography–mass spectrometry (HS-SPME–GC–MS) chromatograms of identification compounds from MP particles extracted from alluvial soil samples, spiked with three concentrations (2 MPs, 5 MPs, and 10 MPs) of each polymer type (PET: dimethyl terephthalate (1a); PS: styrene (2a) and dimer trans(cis)-1,2-diphenylcyclobutane (3a and 4a); PVC: chlorooctane (5a); PP: 4,6-dimethyl 2-heptanone (6a); and PE: eicosane (7a)).

Identification of MPs extracted from spiked alluvial soil and compost samples using density separation with ZnCl_2_ solution was possible via the HS-SPME–GC–MS method. As described in Section 2.4, the proposed method by Šunta et al. [44] was optimised for improved identification of PET. To improve the melting of PET, a thermal decomposition step was carried out in a sand bath, and PET particles were analysed separately from other MP polymers. PS, PVC, and PP in alluvial soil and compost samples were identified with previously proposed characteristic compounds for the identification of MPs after their thermal decomposition: styrene and dimer, chlorooctane, and 4,6-dimethyl 2-heptanone, respectively [39,41,44,49]. In the chromatogram of analysed MP mixtures (PS, PVC, PP, and PE), pentadecane—a characteristic compound proposed for the identification of PE—was not observed; however, the series of higher alkanes (from tetradecane, C_14_, up to eicosane, C_20_) was observed, and the presence of PE was confirmed. PET extracted from compost samples was identified using the previously proposed characteristic compound—dimethyl terephthalate (DMTP) [41,44,49]—while DMTP from analysed PET particles extracted from alluvial soil was not determined, and ethyl methyl terephthalate (signal 1a, Figure 5) at a retention time of 16.994 min was used instead. Ethyl methyl terephthalate was chosen in the case of alluvial soil samples because it is one of the terephthalate derivatives that are produced upon thermal decomposition of PET, according to Yakovenko et al. [50].

**Figure 6:**
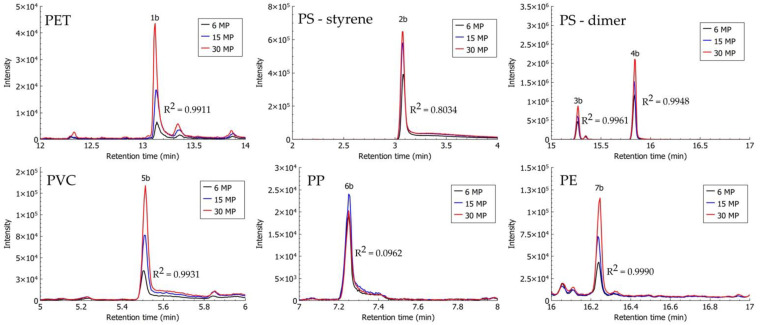
Headspace solid-phase microextraction–gas chromatography–mass spectrometry (HS-SPME–GC–MS) chromatograms of identification compounds from MP particles extracted from compost samples, spiked with three concentrations (2 MPs, 5 MPs, and 10 MPs) of each polymer type (PET: ethyl methyl terephthalate (1b); PS: styrene (2b) and dimer trans(cis)-1,2-diphenylcyclobutane (3b and 4b); PVC: chlorooctane (5b); PP: 4,6-dimethyl 2-heptanone (6b); and PE: eicosane (7b)).

After optimisation of the HS-SPME–GC–MS method for the identification and analysis of spiked samples of soil with different MP contents, the potential of this method for the descriptive quantification of MPs was observed. In a detailed analysis of chromatographic peaks of characteristic compounds for the identification of MPs, a relationship between the number of analysed particles and the signal response (determined area under the chromatographic peak) was observed (Figure 5 and Figure 6). Cumulative samples of three replicates were analysed (6, 15, and 30 MPs).

In the case of alluvial soil samples, a linear relationship between the number of particles and the signal response with R^2^ > 0.97 was observed for ethyl methyl terephthalate (PET), styrene and the dimer—trans(cis)-1,2-diphenylcyclobutane (PS), chlorooctane (PVC), and eicosane (PE) (Figure 5). For 4,6-dimethyl 2-heptanone (PP), the linear coefficient was very high (R^2^ = 0.9997); nevertheless, the signal response (signal 6a in Figure 5)—or area under the chromatographic peak—was not increasing with the number of MPs, as would be expected, but decreasing.

A high linear relationship (R^2^ > 0.99) between the signals of characteristic compounds of individual polymer types of MPs extracted from compost samples on the one hand, and the number of MPs on the other, was also observed in the case of DMTP (PET), trans(cis)-1,2-diphenylcyclobutane (PS), chlorooctane (PVC), and eicosane (PE) (Figure 6). There was a low linear relationship observed for styrene (PS) with R^2^ = 0.8034, and none for 4,6-dimethyl 2-heptanone (PP).

Observed linearity (above R^2^ > 0.98 in alluvial soil, and above R^2^ > 0.80 in compost) in a controlled laboratory environment with established particle size, number, average particle mass, and polymer type demonstrated that the HS-SPME–GC–MS method could be used for tentative descriptive quantification of PET, PS, PVC, and PE in analysed matrices. However, it is a prerequisite that samples are prepared for the identification of MPs using pretreatment methods for the elimination of organic matter and extraction of MP polymers from the matrix. Quantitative measurements with the SPME headspace extraction technique depend on the type of SPME coating, the partitioning coefficient between the fibre coating and the analytes, the chemical properties of the analytes, the complexity of the sample, and temperature, while particle size, mass, and chemical structure (additives) also need to be considered in the identification of MPs [51,52].

The nonlinearity of 4,6-dimethyl 2-heptanone (PP) could be attributed to the possible interactions in the headspace vial between volatile substances emitted from MP polymers during the thermal decomposition step of the identification method. As the best signal response can be observed at the lowest number of analysed PP particles, it is also possible that competition for the active adsorption sites on the SPME fibre occurred between 4,6-dimethyl 2-heptanone and other volatile substances, as was also observed by Dutra et al. [53] and Demets et al. [54]. Therefore, future studies on the quantification of MPs in environmental matrices using HS-SPME–GC–MS would be of interest.

### 3.4. Applicability of the Developed Methods to Environmental Samples

Density separation was applied to real compost samples from municipal organic waste to extract natively present MPs. The procedure was carried out as described in Section 2.3. From 10 g of compost, 36 ± 9 MPs were extracted, with an average mass of 6.3 ± 1.8 mg (*n* = 3). Extracted particles were placed in vials and identified via HS-SPME–GC–MS as described in Section 2.4. The presence of two polymers was determined: PS and PP, the latter being tentatively identified using methylated higher alkanes in the absence of the characteristic compound for PP, i.e., 4,6-dimethyl 2-heptanone. It should be noted, however, that the identification method has currently only been developed for the five aforementioned plastic polymers (PET, PVC, PE, PS, and PE), while the determination of characteristic compounds for other plastics is underway. Hence, the detection of other potentially present MP polymers was not possible.

Biowaste compost is frequently contaminated with MPs, originating from incompletely removed plastic bags, plastic packaging of food, incompletely degraded biodegradable plastics, and other sources [16]. A study quantifying MPs in organic fertilisers found between 20 and 24 MPs (>1 mm) per kg of compost [17], which is 150–180 times less than the amount isolated in this study; the employed method for MP extraction, however, was different (wet sieving), which could, in part, explain the differences in MP numbers. Other studies reported up to 2800 MPs per kg of compost, which more closely aligns with the abundance reported herein (2900–4600 MPs per kg) [55].

The strengths of the methods reported in this study include high MP recoveries from soil and compost via the density separation method, as well as the methods being easy to handle and widely accessible. The identification method has the advantage of simultaneous multi-polymer analysis and the use of common analytical equipment. There are, however, some drawbacks: Neither method was optimised for large sample processing (e.g., >1 kg). Additionally, the HS-SPME–GC–MS method is not yet developed for the identification of other plastic polymers, e.g., PA and biodegradable plastic polymers. Furthermore, the presence of organic matter in the MP isolate hinders polymer identification, as the limit of detection (LOD) rises with increasing organic matter content; therefore, efforts to significantly reduce organic matter should be made prior to sample analysis.

## 4. Conclusions

Two methods for the extraction of MP particles were tested and optimised: oil-based extraction, and density separation. Both methods achieved high recoveries of spiked MP polymers of low and high density in alluvial soil; however, in compost, density separation with ZnCl_2_ solution proved to have better recoveries. In addition, considering other factors—such as time efficiency, ease of handling, and the potential for scaling up—density separation was shown to be the better option. Additionally, oxidation before and after extraction was necessary in order to considerably reduce the organic matter content of compost samples. Identification of MPs via the HS-SPME–GC–MS method was carried out successfully via the detection of characteristic compounds for each polymer type. The potential to use this method for descriptive quantification was proven by a linear relationship between the number of particles and the signal response on chromatograms in the case of PET, PS, PVC, and PE. Density separation was employed to isolate natively present MPs from municipal biowaste compost (36 ± 9 MPs per 10 g). The presence of PS and PP was confirmed with HS-SPME–GC–MS.

This study contributes towards the understanding of the suitability of available extraction methods for soil and compost samples, and additionally presents the possibility of using commonly available analytical equipment for MP identification.

## Figures and Tables

**Table 1:** Plastic polymers used, their source, and their properties.

Polymer Type (1–2 mm)	Source	Density (g cm^−3^)	Melting Temperature (°C)
PP	Pellets (Golias Ltd., Ljubljana, Slovenia)	0.85–0.88	179
PE	Bottle cap (Radenska)	0.91–0.96	108–141
PS	Pellets (Golias Ltd.)	1.04–1.10	242–276
PVC	Blister pack	1.38–1.40	220–305
PET	Plastic bottle (Radenska)	1.33–1.48	264

PP: polypropylene; PE: polyethylene; PS: polystyrene; PVC: polyvinyl chloride; PET: polyethylene terephthalate.

**Table 2:** Comparison of recoveries of PET and LDPE microplastic particles, achieved in two extraction methods: oil-based extraction in three different vessels, and density separation. *n* = 6; results are presented as mean ± standard deviation (SD).

Type of Soil	No. and Type of MPs	Oil-Based Method (%)			Density Separation (%)
		PTFE Cylinder	Glass Cylinder	Plastic Syringe	
**Alluvial soil**	10 PET	96.7 ± 8.2	100.0 ± 0.0	96.7 ± 5.2	93.3 ± 5.2
	20 PET	97.5 ± 2.7	97.5 ± 2.7	97.5 ± 4.2	99.2 ± 2.0
	10 LDPE	98.3 ± 4.1	98.3 ± 4.1	98.3 ± 4.1	98.3 ± 7.5
	20 LDPE	96.7 ± 8.2	96.7 ± 4.1	97.5 ± 2.7	100.0 ± 0.0
**Compost**	10 PET	N.A. ^1^	N.A. ^1^	80.0 ± 11.0	98.3 ± 4.1

^1^ Not applicable.

## Data Availability

The data presented in this study are available in the Zenodo digital repository at https://doi.org/10.5281/zenodo.5607477 (accessed on 21 November 2021).
